# Supplementary material for: Intermittent Lactobacilli-containing Vaginal Probiotic or Metronidazole Use to Prevent Bacterial Vaginosis Recurrence: A Pilot Study Incorporating Microscopy and Sequencing
Source: Sci Rep. 2020 Mar 3;10:3884. doi: 10.1038/s41598-020-60671-6 (PMC7054572; doi:10.1038/s41598-020-60671-6)
Supplement: Supplementary file 1 — VMB Safety Efficacy Supplement1. [file 41598_2020_60671_MOESM1_ESM.pdf]

Intermittent Lactobacilli-containing Vaginal Probiotic or Metronidazole Use to Prevent Bacterial Vaginosis Recurrence: A Pilot Study Incorporating Microscopy and Sequencing

Authors: Janneke H.H.M. van de Wijgert, Marijn C. Verwijs, Stephen Agaba, Christina Bronowski, Lambert Mwambarangwe, Mireille Uwineza, Elke Lievens, Adrien Nivoliez, Jacques Ravel and Alistair C. Darby.

SUPPLEMENT 1

Recruitment
The target population was women at high risk of urogenital infections living in Kigali, Rwanda. Recruitment activities were implemented by study staff with the help of Community Mobilizers, who were selected due to their strong connections with high-risk women in Kigali. They helped staff organize recruitment meetings in relevant communities. At these meetings, an anonymous pre-screening checklist containing the most important eligibility criteria was used, but no information recorded, and potentially eligible women were referred to the Rinda Ubuzima research center for screening.

Eligibility criteria
The main eligibility criteria are described in the manuscript. We focused on BV patients, but also included women diagnosed with TV because we expected most of them to also have BV (which was indeed the case), and because BV and TV are treated with an identical metronidazole regimen. Additional exclusion criteria included physician-observed genital ulcers, condylomata or other genital abnormalities; having had an invasive gynecological procedure in the three months prior to screening; history of significant urogenital prolapse, undiagnosed vaginal bleeding, urine or fecal incontinence, or blood clotting disorders; allergy to metronidazole or any other components of the study drugs; not willing to terminate use of other oral or vaginal probiotics; or participating in another health intervention study. These criteria applied to only six women (Figure 1). HIV-positive and pregnant women were referred to local HIV and antenatal care clinics for care.

Informed consent procedures
All participants provided written informed consent. The age of majority for Rwandan women was 21 at the time of study implementation, and we therefore also obtained parent/guardian consent for non-married participants aged 18-20 years. Participants and/or parents/guardians with insufficient literacy could sign by thumbprint but the informed consent process was observed by an independent witness who co-signed the informed consent form. The witness could not be a Rinda Ubuzima staff member, but could be another participant. Participants received the equivalent of three GBP per visit in local currency as a reimbursement for time spent at the clinic and transport costs.

Clinic procedures
An overview of visit procedures and samples collected is presented in Figure 1. At the screening visit, participants underwent a face-to-face interview, speculum examination, real-time testing for HIV, pregnancy, urinary tract infection, BV, and TV, and collection of samples for sexually transmitted infection and future molecular testing (see below). At the end of this visit, preliminary eligibility was assessed, and initial treatments and referrals were given based on available test results. Testing for *Chlamydia trachomatis*, *Neisseria gonorrhoeae*, *Treponema pallidum* (syphilis), and herpes simplex virus type 2 was conducted on stored samples after the participant had left the clinic. Results of the syphilis and herpes simplex type 2 tests were shared with participants at a results visit one to two weeks after the screening visit, additional treatments were given as required, and eligibility was reassessed. Positive herpes simplex type 2 serology was not a reason for exclusion. Chlamydia and gonorrhea results were shared with participants as soon as they were available. We did not use them to determine eligibility as originally intended because the turn-around time of polymerase chain reaction (PCR) testing at the National Reference Laboratory in Kigali was slow. At the end of the screening and results visits cascade, women were either declared ineligible, or diagnosed with BV (by Nugent score[1] and/or modified Amsel criteria[2], defined as two of three positive: vaginal pH>4.5, positive whiff test, or ≥20% clue cells) and/or TV

(by culture and/or wet mount) and treated with 500 mg oral metronidazole twice per day for seven days. An enrollment visit was scheduled within three days after treatment completion, and only women whose treatment had been successful (no BV by modified Amsel criteria and no TV on wet mount), and who were still not pregnant and free of urinary tract infection and syphilis, were randomized to the four groups described in the manuscript. Women could be rescreened a maximum of three times.

The Chief Investigator (JvdW) based in Liverpool used a random number generator to assign participant identification numbers to groups in blocks of four. At the Rinda Ubuzima clinic, each eligible woman was assigned the next available participant identification number, and the corresponding sealed envelope was opened to reveal her randomization group. The laboratory technicians were blinded but it was not possible to blind the clinicians and participants. Behavioral counseling was offered to all participants in all randomization groups. The counseling focused on reducing known risk factors for BV such as unprotected sex, vaginal hygiene practices, male partner penile hygiene practices, and alcohol use (which can lead to unprotected sex and can cause severe side effects when used in combination with metronidazole). At follow-up visits, participants underwent a face-to-face interview, counselling, speculum examination, laboratory assessments (BV by modified Amsel and Nugent criteria, TV by wet mount and culture, vulvovaginal candidiasis by wet mount, and additional testing if clinically indicated), and sampling for future molecular testing. HIV, sexually transmitted infection, urinary tract infection, and pregnancy tests were repeated at M6 only. All participants were offered male condoms free of charge at each study visit. Forty six participants made unscheduled visits to collect treatment for an infection that was diagnosed by laboratory testing after the participant had left the clinic (n=35 women), because of new symptoms (n=14), and/or to withdraw informed consent (n=1).

Dosing and adherence

The dosing regimens were based on earlier studies (metronidazole) and on marketing approvals (vaginal probiotics), except that GynLP was used every four days for two months instead of the three weeks recommended by the company so that all women in the intervention groups dosed for two months. We had initially intended to use the Sobel et al regimen of a metronidazole gel,[3] but we were not able to identify any metronidazole gels that had proven stability at 25°C and 30°C for up to two years. Given that the trial was conducted in Rwanda, the University of Liverpool Sponsorship Committee required proven stability at these temperatures, and we ended up using metronidazole tablets that did have proven stability. The study products were stored in an air-conditioned room with controlled access until handed to participants. Adherence to the interventions was assessed at the D7, M1, and M2 visits by structured interviewer-administered questionnaire, review of a diary card that the participant completed in between study visits, review of returned used packaging and unused product, and by asking the participant to complete a self-rating adherence scale. These different sources of adherence data were triangulated to arrive at an overall level of adherence (between 0-100%) for each participant between visits. Women were allowed to cease product use during menstrual bleeding (done by 12 women), and the adherence data were not adjusted for this.

In the probiotic strains detection discussion in the manuscript, we state that we estimate the average total bacterial load of the vagina to be in the order of $2x10^{10}$ bacteria. This is based on the following assumptions. The average vaginal surface area was estimated to be 87.5 cm^2.[4] One Dacron swab head in this study absorbed about $10^6/\mu L$ bacteria. If we assume that one swab head absorbs on average 200 µL,[5] and that this covers about one cm^2 of a total of

about 100 cm^2 vaginal surface, the total bacterial load in the vagina would be in the order of $2x10^{10}$ bacteria.

Diagnostic testing

All diagnostic testing was conducted at Rinda Ubuzima or the National Reference Laboratory in Kigali using validated procedures. Vaginal swab, blood, and urine specimens were processed on the collection day and either tested immediately or stored at -80°C until testing. Dacron vaginal swabs were used for wet mounts, Gram stains, and TV InPouch culture (Biomed Diagnostics, White City, OR, USA) at all study visits, as described in the manuscript. All other diagnostic tests were only done at screening, M6, and when judged clinically necessary by the physician, with the exception of pregnancy and urinalysis tests, which were repeated at enrollment prior to randomization. Whole blood was tested for HIV 1/2 using the Kehua HIV Rapid Test (Kehua Bio-engineering, Shanghai, China), followed by the Alere Determine HIV-1/2 Rapid Test (Abbott Laboratories, Tokyo, Japan) for confirmation of positive results and the Unigold HIV Rapid Test (Trinity Biotech, Bray, Ireland) as tie-breaker (if applicable). Urine was tested for pregnancy using an hCG dipstick test and urinary tract infection using a urinalysis dipstick test (both by Nova, Atlast Link Technology, Beijing, China). Plasma was tested for herpes simplex type 2 serology (Kalon, Guildford, UK; using an optical density cut-off of >1.1 for a positive result and <0.9 for a negative result) and syphilis by Rapid Plasma Reagin test followed by *Treponema pallidum* Hemagglutination Assay (both by Spinreact, Girona, Spain). Endocervical swabs were tested for *C. trachomatis* and *N. gonorrhoeae* by real-time PCR (Presto, Beek, The Netherlands).[6] A sub-sample of 49 M6 samples were tested by GeneXpert CT/NG assay (Cepheid, CA, USA) after study completion. The sensitivities and specificities of the two real-time PCR assays are high and comparable.[7]

Molecular laboratory methods

DNA extraction

For molecular testing, the physician collected two Dacron vaginal swabs per woman during speculum examinations at screening, enrollment, and each scheduled follow-up visit (N=1,016 physician-sampled swabs). The small discrepancy between expected ([176+74+65+66+66+64] x2=1,022; Figure 1) and collected physician-sampled swabs can be explained as follows: six of the 176 women who attended a screening visit did not provide swabs, four of the 68 randomized women missed at least one study visit, and a few extra swabs were taken from women who attended an enrollment visit but turned out to be ineligible for randomization and from two randomized women. The swab heads were stored dry at Rinda Ubuzima at -80°C on the collection day. The 12 women in the self-sampling group self-sampled two flocked swabs (Copan Diagnostics, Murrieta, CA, USA) every Monday, Wednesday, and Friday of the first month after randomization (N=258 self-collected swabs). The discrepancy between expected (12x12x2=288) and collected self-collected swabs can be explained as follows: one of the 12 women completed the study but never self-sampled, and the remaining 11 women combined missed one self-sampling time point and sampled one instead of two swabs at four time points. The swab heads were initially stored at room temperature in the participant's home in cryovials containing 1 ml RNALater (Thermo Scientific, Paisley, UK), and then at Rinda Ubuzima at -80°C within seven days after collection. Frozen samples were shipped to Liverpool on dry ice. DNA extraction and sequencing were done at the University of Liverpool Centre for Genomic research.[8] DNA was extracted from one sample per participant per time point (N=639 swabs). Those frozen dry were thawed. Those frozen in RNALater were thawed and then centrifuged at 16,000 g for 10 minutes at 4 °C: supernatants were discarded, and pellets and swab heads were used for DNA

extraction. DNA was extracted from each sample by adding 180 µl of enzymatic lysis buffer containing lysozyme (Sigma-Aldrich, Dorset, UK); incubation for 30 minutes at 37 °C; adding 25 µl of proteinase K and 200 µl of buffer AL using the Qiagen DNeasy Blood and Tissue kit (Qiagen, Manchester, UK); incubation for 30 minutes at 56 °C; and bead-beating after adding 200 mg of 0.1 mm zirconia/silica beads (Thistle Scientific, Glasgow, UK) on a Qiagen TissueLyser II (Qiagen, Manchester, UK) for 5 minutes at 25 Hz. Next, 200 µl of 100% ethanol was added, the sample was centrifuged, the swab head was discarded, and the pellet was purified in four subsequent centrifugation steps after adding one-by-one 200 µl 100% ethanol, 500 µl buffer AW1, 500 µl buffer AW2 and 75 µl buffer AE as per manufacturer's instructions (Qiagen, Manchester, UK). We included one negative control (an empty tube) with each DNA extraction round of 24 study samples to be able to detect contaminants in extraction reagents downstream. The DNA concentration of randomly selected samples was measured by Qubit (Invitrogen, Thermo Scientific, Paisley, UK) and the DNA quality of all samples by Nanodrop (Thermo Scientific, Paisley, UK).

PCR amplification and 16S rRNA sequencing
Each of the 667 DNA samples (639 study samples and 28 negative controls) underwent two PCR rounds for 16S rRNA gene amplification and barcoding. In the first PCR round, the V3-V4 region of the 16S rRNA gene was amplified as described previously.[9] DNA was amplified in a 25 µl reaction volume using 1.25 µl of a 10 µM concentration of 319F 5'-ACTCCTACGGGAGGCAGCAG-3' forward primer and 1.25 µl of a 10 µM concentration of 806R 5'-GGACTACHVGGGTWTCTAAT-3' reverse primer, 12.5 µl NEB Next HF 2x PCR Master Mix (New England Biolabs, Hitchin, UK), 9 µl of nuclease-free water and 1 µl of DNA extraction product. The first denaturation cycle took 30 seconds at 98 °C and was followed by 10 cycles consisting of a denaturation cycle of 10 seconds (at 98 °C), an annealing cycle of 30 seconds (at 58 °C), an extension cycle of 30 seconds (at 72 °C), and a final extension cycle of 5 minutes at 72 °C. PCR products were then purified and size-selected using Agencourt AMPure XP beads (Beckman Coulter, High Wycombe, UK) in a 0.8:1.0 bead-to-sample ratio. The second PCR round was to barcode V3-V4 sequences by a dual-index approach using standard Illumina Nextera XT index kit v2 (Illumina, San Diego, CA, USA), permitting multiplexing of up to 384 samples. The barcoding was performed in a 25 µl reaction volume using 2.5 µl of Index 1 primer, 2.5 µl of Index 2 primer, 12.5 µl NEB Next HF 2x PCR Master Mix and 7.5 µl sample. The first denaturation cycle took 3 minutes at 98 °C and was followed by 15 cycles consisting of a denaturation cycle of 30 seconds (at 98 °C), an annealing cycle of 30 seconds (at 55 °C), an extension cycle of 30 seconds (at 72 °C), and a final extension cycle of 5 minutes at 72 °C. PCR products were then purified using AMPure beads as explained above, again in a 0.8:1.0 bead-to-sample ratio. We added a negative control to each PCR run (10 µl of nuclease-free water instead of 9 µl of nuclease-free water and 1 µl of DNA) to identify contaminants, as well as a commercially available positive control (10 µl of 0.2 ng/µl ZymoBiomics Microbial Community DNA standard; Zymo Research Corp, Irvine, CA, USA). The DNA extraction negative controls were also included in the PCR runs. DNA from samples collected at different visits but belonging to the same participant were included in the same PCR run to control for inherent differences between PCR runs. PCR product DNA concentrations of each sample (N=683: 639 study samples, eight positive PCR controls, eight negative PCR controls, and 28 negative DNA extraction controls) were measured using the Qubit Fluorometer with the dsDNA HS Assay kit (Invitrogen, Thermo Scientific, Paisley, UK). All samples, including the positive and negative controls, were successfully amplified and used for subsequent steps.

Amplicons from samples were evenly pooled into sequencing libraries at a mass of 0.8 ng

DNA per amplicon. To achieve this, Qubit DNA concentrations were used to calculate the volumes of each study sample to be added. Samples with a DNA concentration of <0.30 ng/µl (e.g., the negative controls) were added in a fixed volume of 1 µl. The two libraries were sequenced on an Illumina HiSeq instrument (Illumina, San Diego, CA, USA), run in rapid mode, 2x300bp using a 250PE and 50PE kit. DNA from samples collected at different visits but belonging to the same participant were included in the same library (and hence, sequencing run) to control for inherent differences between sequencing runs.

Panbacterial 16S rRNA gene qPCR

The panbacterial 16S rRNA gene copy concentrations of all samples collected at study visits and containing at least 1,111 reads (rarefaction depth) by Illumina HiSeq sequencing (N=393; see 'Further data processing' below for rationale) were determined at the Institute for Genome Sciences of the University of Maryland (Baltimore, MD, USA) using the BactQuant qPCR assay. This assay was developed based on an analyses of 4,938 16S rRNA gene sequences in the Greengenes database.[10,11] The DNA samples were tested as described previously.[11,12] Briefly, 1.5 µl of template (1:10 diluted DNA) was added to 3.5 µl of reaction mix, with the final reaction containing 1.8 µM each of the forward (341F) and reverse (806R) primer targeting the 16S V3-V4 region, 225 nM of the TaqManW probe, 1X Platinum Quantitative PCR SuperMix-UDG with ROX (Invitrogen, Thermo Scientific, Waltham, MA, USA) and molecular-grade water. Each experiment included an in-run standard curve (ranging from 10 to 10^8, with 10^2–10^8 in 10-fold serial linear dilutions) and no-template controls performed in triplicate. Amplification and real-time fluorescence detections were performed on the Bio-Rad CFX 384 instrument (Bio-Rad Inc., Hercules, CA, USA) using the following PCR conditions: three minutes at 50 °C for UDG treatment, 10 minutes at 95 °C for Taq activation, 15 seconds at 95 °C for denaturation and one minute at 60 °C for annealing and extension, times 40 cycles. Cycle threshold (Ct) value for each 16S qPCR reaction were obtained using a manual Ct threshold of 0.05 and automatic baseline. The 16S rRNA gene concentration was reported in copies/µL for each sample.

Molecular data processing

We obtained a mean raw unpaired read count of 374,543 reads per study sample (95% confidence interval (CI): 305,845 – 443,242) in run 1 and 302,431 reads per study sample (95% CI: 266,423 – 338,440) in run 2. Reads were first demultiplexed, and primer sequences were removed from forward and reverse reads using Cutadapt 1.2.1.[13] All subsequent steps were performed in the DADA2 v1.4.0 package for large paired end datasets in R v3.4.3 (R core team, 2015).[14] We chose DADA2 because of its ability to resolve reads differing by only one nucleotide, maximizing our chances of differentiating probiotic reads from naturally occurring *Lactobacillus* species reads. Error correction was performed using the *fastqFilter* command with parameter settings aiming to maximize read retention. For forward and reverse reads, respectively, the minimum read lengths (*truncLen*) were set to 260 and 210 based on the quality plots, *maxEE* to a maximum of 5 and 8 expected errors, *maxN* to zero ambiguous bases allowed, and *truncQ* to zero. Around 10% of reads were discarded after error correction. Next, error rates of forward and reverse reads were determined using the *learnErrors* command. Forward and reverse reads were dereplicated (assigned to unique amplicon sequence variants (ASVs)) using the *derepFastq* command, and denoised (ASVs with higher than average error rates discarded) using the *dada* command.[14,15] Forward and reverse reads were then merged into overlapping reads using the *mergePairs* command. Bimeras (chimeric compositions of two separate parent ASVs) were removed using the *removeBimeraDenovo* command with the Silva v128 database as the reference database;[16] 10.1% of ASVs were identified as bimeric and removed. Overall, a median of 16% of the raw

reads per study sample was removed during these DADA2 clean-up steps.

Taxonomic assignment was also done in DADA2 in two steps: *assignTaxonomy* to map ASVs to taxa at genus level or above using the RDP classifier with a minimum bootstrap value of 50% and the Silva v128 database as the reference database,[16,17] followed by *addSpecies* to map ASVs to species level, allowing only ASVs with exact (100%) matches with species in the Silva database to be assigned to that species, and allowing assignment of one ASV to multiple species.

Further data processing
Further data processing was performed in Microsoft Excel 2013 (starting with a spreadsheet containing the sequences, taxonomic assignments, and read counts for each ASV per sample) and STATA v13 (StataCorp, College Station, TX, USA). We removed all rare ASVs (defined as a read count in all samples combined of less than 100), four non-bacterial ASVs, and two likely contaminant ASVs (a *Rhodanobacter glycinis/terrae* and a *Sneathia* genus) that were present in more than one negative control at relative abundances higher than in any study sample. The vaginal taxa BV-associated bacterium 1 (BVAB1), BVAB2, *Mageeibacillus indolicus* (BVAB3), BVAB TM7 and *Fenollaria massiliensis* are not included in the Silva v128 database but their sequences have been published elsewhere. Similarly, the *Lactobacillus* and *Bifidobacterium* species included in the vaginal probiotic Ecologic Femi+ (EF+; Winclove Probiotics, Amsterdam, Netherlands) are not included in the Silva database, but their sequences were provided to us by Winclove. The vaginal probiotic Gynophilus LP (GynLP; Biose, Arpajon-sur-Cère, France) contains *Lactobacillus rhamnosus* (Lcr strain 35), and the NCBI database contains a reference sequence for this probiotic strain.[18] We identified all of the above species in our ASV spreadsheet using the *Needleman-Wunsch Global Align Nucleotide Sequences* function on the NCBI website,[19] requiring 100% matches between reference sequences and uploaded DADA2-derived ASVs of interest. The Silva-based taxonomic assignment of 146 ASVs with a relative abundance of at least 0.05% of the read count of all samples combined (out of a total of 1,797 ASVs) were double-checked using the *Microbial Nucleotide BLAST (BLASTn)* function on the NCBI website[20,21] using the non-redundant V3-V4 version of the Vaginal 16S rDNA Reference Database as a tiebreaker,[22] three discordances were resolved, 24 *Lactobacillus* genus ASVs were reassigned to various *Lactobacillus* species, six *Gardnerella* genus ASVs were reassigned to *G. vaginalis*, and one *Atopobium* genus ASV was reassigned to *A. vaginae*. Next, read counts for ASVs assigned to the exact same taxonomy were summed for each sample, except for ASVs assigned to probiotic strains. Finally, we rarefied at a depth of 1,111 reads (the lowest total read count for a specific sample above 1,000 reads) using the *GUniFrac* 1.0 package in R.[23] The rarefied ASV table contained 629 samples and 401 unique ASVs (10/639 samples became invalid due to rarefaction), with 255 (63.6%) mapping to species level, 116 (28.9%) to genus level, and 30 (7.5%) to higher taxonomic levels. Rarefied read counts were transformed into relative abundances using the *prop.table* function in R. Of the 401 ASVs, 177 ASVs were present at a relative abundance of at least 1% in at least one sample; the other 224 ASVs were minority species.

Of the 393 samples that were tested by BactQuant assay (Figure 1), five did not amplify in two of three, or all three, of the triplicate reactions and were considered invalid. The 16S rRNA gene concentration results of the other samples were normally distributed with the exception of nine samples with a concentration of <1,000 copies/μl, which were considered outliers. A total of 14/393 samples (= 3.6%) were therefore excluded from all analyses. We estimated the ASV-specific concentrations per sample using the sample-specific 16S rRNA

gene concentration data. First, we identified the 16S rDNA gene copy number per ASV in the NCBI version of the rrnDB database,[24] and in the case of missing data, in the RDP version of the rrnDB database (which only contains information at genus level and above) and the Greengenes database.[10] If an ASV was mapped to multiple species at genus level, the mean of the mean 16S gene copy number for each individual species was used. If the mean 16S gene copy number of a species was not present in the database, we used the mean copy number of the corresponding genus. BVAB1 and BVAB2 belong to the *Clostridiales* order and lower level taxonomic information is not available. We therefore used the *Clostridiales* order copy number (=4.62). Concentrations in cells/µl per ASV per sample were estimated by multiplying the ASV-specific copy-normalized rarefied relative abundance by the sample-specific 16S rRNA gene copies concentration. This method has been shown by others to correlate well with species-specific quantitative PCR results for non-minority species.[25,26] This yielded concentrations for 401 ASVs in 379 samples in cells/µl, which were $\log_{10}$-transformed. Concentrations between zero and one cell/µl were set to one prior to $\log_{10}$-transformation to prevent skewed negative values.

Trial endpoints and hypotheses

The primary aims of the trial were to determine the safety and preliminary efficacy of the interventions, each compared to the no intervention group. We hypothesized that all interventions would be safe. The primary preliminary efficacy endpoints were the incidence of BV by Nugent score and modified Amsel criteria (which were hypothesized to decrease), and symptomatic vulvovaginal candidiasis (VVC) by wet mount (which was hypothesized not to increase). The secondary preliminary efficacy endpoints included membership of specific VMB clusters/types and bacterial group concentrations over time as determined by Illumina HiSeq sequencing.

Safety endpoints

The main safety endpoints were self-reported solicited and unsolicited (serious) adverse events (AEs) and social harms, and clinician-observed speculum exam findings. Adverse events were coded using the Medical Dictionary for Regulatory Activities (medDRA v19.1, McLean, VA, USA). Laboratory test results were not considered AEs, but primary and secondary endpoints.

Primary preliminary efficacy endpoints

For the BV by Nugent 7-10, modified Amsel criteria and VVC endpoints, see the 'diagnostic testing' section above. Analyses were also conducted using a Nugent score of 4-10 and the full Amsel criteria as the definition of BV but the results are not shown because they are similar to the Nugent 7-10 and modified Amsel criteria results, and less informative than the molecular data.

Molecular endpoints: richness, diversity, VMB types, and bacterial groups

Data reduction was required for molecular efficacy analyses, and was done in three different ways. First, we calculated richness and alpha diversity for each sample using the rarefied relative abundance data. Richness was defined as the total number of ASVs per sample. Alpha diversity was determined by Simpson diversity (1-D) using the *phyloseq* package v1.14.0 in R.[27] Second, we used existing knowledge to assign all 401 ASVs to four bacterial groups, as described in the manuscript and in Supplement 2. Third, we clustered the rarefied relative abundance data using the *phyloseq* package in R using Euclidean distance with complete linkage.[27] We used this information, and information from previously conducted studies, to classify samples into eight mutually exclusive VMB types (each sample was

assigned to only one VMB type) as described in the manuscript. To improve the statistical power of the mixed effects models, and to facilitate visualizations of VMB transitions in alluvial diagrams, the eight VMB types were condensed further into four 'pooled VMB types' as described in the manuscript.

Statistical analyses
Statistical analyses were performed using STATA v13 (StataCorp, College Station, TX, USA). They are described in the manuscript, and additional technical details are available here. We used the *phyloseq* and *gplots* packages in R to make heatmaps of the 20 ASVs with the highest mean relative abundance (Figure S1A) or concentration (Figure S1B) across all study samples.[28] Bar graphs and line graphs were made using the *catplot* and *scatter* functions in STATA and alluvial diagrams were made using the *ggalluvial* package in R.[29] For cross-sectional analyses, we used Fisher's exact test to compare binary and categorical variables, Kruskal-Wallis test to compare continuous variables, and Mann Whitney U test for pairwise comparisons of continuous variables if the Kruskal-Wallis test was significant at $p<0.05$. For longitudinal analyses, we used incidence rates (IRs) and incidence rate ratios (IRRs) with 95% confidence intervals (CI; primary endpoints only), and mixed effects models. IRs were defined as the number of new infections during follow-up divided by the person-years at risk for that infection using the *ststet* function in STATA. Women who had BV recurrence within 10 days that persisted until M6 were considered persistent infections to prevent inflation of the IRs. IRRs were calculated by dividing the IR of each intervention group by the IR of the control group. Mixed effects models were done in STATA using the *xtmelogit* function for categorical endpoints and the *mixed* function for continuous endpoints. All models included one VMB endpoint at a time as the outcome, the participant identification number as the random effect, and randomization group as the main fixed effect. Three variables were added as additional fixed effects in adjusted mixed effects models because they were associated at $p<0.05$ in mixed effects models with at least one of four a priori selected VMB endpoints (Nugent score, or concentrations of lactobacilli, BV-anaerobes, or pathobionts; Table S6), and data were available at all study visits. They were 'current use of hormonal contraception or being pregnant' (versus not using contraception and not being pregnant; six samples from copper intrauterine device users were excluded), a 'sexual risk' composite variable (with lower risk defined as having reported condom use at each vaginal sex act since the last study visit and fewer than the median of five sex partners in the past month), and 'age' (30 years or older versus younger than 30 years; the median age of the screened population was 30 years). One additional variable was associated with one of the a priori selected VMB outcomes at $p<0.05$: managing menses with sanitary pads versus other methods (Table S6). However, this variable was not added as an additional fixed effect in the adjusted mixed effects models due to only having been recorded at enrollment visits and not at follow-up visits.

All analyses were conducted on the intent-to-treat (ITT) population (n=68), and IR and IRR analyses were also conducted on a modified ITT population (n=51). Women who were BV-negative by modified Amsel criteria but BV-positive by Nugent score at the time of randomization (n=17) were excluded from the modified ITT population. Another 17 women had ongoing chlamydia and/or gonorrhea infection at the time of randomization, but the molecular analyses did not identify substantial differences in the VMB compositions of women with and without ongoing infection and we therefore did not exclude them (Figure S2). In accordance with local treatment guidelines, BV and VVC were treated when laboratory-confirmed and symptoms and/or clinician-observed signs were present. TV and other sexually transmitted infections were always treated when identified by laboratory

testing. Some women received antimicrobial drugs for other ailments from external clinics. Antimicrobial use during the study is shown in Table S2. We took antimicrobial use into account to determine whether cases were incident or persistent, but we did not remove users from the modified ITT population because they were evenly distributed between the randomization groups and these short course treatments were expected to have much less of an effect on the VMB than the longer-term interventions.

IR and IRR analyses were conducted for the product use period (between enrollment and M2, including samples from D7, M1, and M2) as well as the period between M2 and M6 (M6 samples). Mixed effects models were done for the product use period only (D7, M1, M2, and self-collected samples) to determine if any of the observed effects were statistically significant; we did not run mixed effects models for the period after product cessation because the IR/IRR analyses and graphs had already shown that the observed effects during the interventions had disappeared after cessation of the interventions.

References

1. Nugent, R. P., Krohn, M. A. & Hillier, S. L. Reliability of diagnosing bacterial vaginosis is improved by a standardized method of gram stain interpretation. *J. Clin. Microbiol.* **29**, 297–301 (1991).
2. Amsel, R. *et al.* Nonspecific vaginitis. Diagnostic criteria and microbial and epidemiologic associations. *Am. J. Med.* **74**, 14–22 (1983).
3. Sobel, J. D. *et al.* Suppressive antibacterial therapy with 0.75% metronidazole vaginal gel to prevent recurrent bacterial vaginosis. *Am. J. Obstet. Gynecol.* **194**, 1283–1289 (2006).
4. Pendergrass, P. B., Belovicz, M. W. & Reeves, C. A. Surface area of the human vagina as measured from vinyl polysiloxane casts. *Gynecol. Obstet. Invest.* **55**, 110–113 (2003).
5. Jespers, V., Francis, S. C., van de Wijgert, J. & Crucitti, T. Methodological issues in sampling the local immune system of the female genital tract in the context of HIV prevention trials. *Am. J. Reprod. Immunol.* **65**, 368–376 (2011).
6. Cuylaerts, V. *et al.* Implementation and evaluation of the Presto combined qualitative real-time assay for *Chlamydia trachomatis* and *Neisseria gonorrhoeae* in Rwanda. *Afr. J. Lab. Med.* **8**, 739 (2019).
7. Gaydos, C. A. Review of use of a new rapid real-time PCR, the Cepheid GeneXpert® (Xpert) CT/NG assay, for *Chlamydia trachomatis* and *Neisseria gonorrhoeae*: results for patients while in a clinical setting. *Expert Rev. Mol. Diagn. Expert Rev. Mol. Diagn.* **14**, 135–137 (2014).
8. Gill, C., van de Wijgert, J. H. H. M., Blow, F. & Darby, A. C. Evaluation of lysis methods for the extraction of bacterial DNA for analysis of the vaginal microbiota. *PLoS One* **11**, e0163148 (2016).
9. Fadrosh, D. W. *et al.* An improved dual-indexing approach for multiplexed 16S rRNA gene sequencing on the Illumina MiSeq platform. *Microbiome* **2**, 6 (2014).
10. DeSantis, T. Z. *et al.* Greengenes, a chimera-checked 16S rRNA gene database and

workbench compatible with ARB. *Appl. Environ. Microbiol.* **72**, 5069–5072 (2006).

11. Liu, C. M. *et al.* BactQuant: an enhanced broad-coverage bacterial quantitative real-time PCR assay. *BMC Microbiol.* **12**, 56 (2012).
12. Nowak, R. G. *et al.* Higher levels of a cytotoxic protein, vaginolysin, in *Lactobacillus*-deficient community state types at the vaginal mucosa. *Sex. Transm. Dis.* 1 (2017).
13. Martin, M. Cutadapt removes adapter sequences from high-throughput sequencing reads. *EMBnet.journal* **17**, 10–12 (2011).
14. Callahan, B. J. *et al.* DADA2: high resolution sample inference from Illumina amplicon data. *Nat. Methods* **13**, 581–583 (2016).
15. Rosen, M. J., Callahan, B. J., Fisher, D. S. & Holmes, S. P. Denoising PCR-amplified metagenome data. *BMC Bioinformatics* **13**, 283 (2012).
16. Pruesse, E. *et al.* SILVA: a comprehensive online resource for quality checked and aligned ribosomal RNA sequence data compatible with ARB. *Nucleic Acids Res.* **35**, 7188–7196 (2007).
17. Wang, Q., Garrity, G. M., Tiedje, J. M. & Cole, J. R. Naïve bayesian classifier for rapid assignment of rRNA sequences into the new bacterial taxonomy. *Appl. Environ. Microbiol.* **73**, 5261–5267 (2007).
18. Coudeyras, S., Marchandin, H., Fajon, C. & Forestier, C. Taxonomic and strain-specific identification of the probiotic strain *Lactobacillus rhamnosus* 35 within the *Lactobacillus casei* group. *Appl. Environ. Microbiol.* **74**, 2679–2689 (2008).
19. Needleman-Wunsch alignment of two nucleotide sequences. Available at: https://blast.ncbi.nlm.nih.gov/Blast.cgi?PAGE_TYPE=BlastSearch&PROG_DEF=blastn&BLAST_PROG_DEF=blastn&BLAST_SPEC=GlobalAln&LINK_LOC=BlastHomeLink (Accessed: 9 December 2019).
20. Altschul, S. F., Gish, W., Miller, W., Myers, E. W. & Lipman, D. J. Basic local alignment search tool. *J. Mol. Biol.* **215**, 403–410 (1990).

21. BLAST: Basic Local Alignment Search Tool. Available at: https://blast.ncbi.nlm.nih.gov/Blast.cgi (Accessed: 9 December 2019).
22. Fettweis, J. M. *et al.* Species-level classification of the vaginal microbiome. *BMC Genomics* **13 (Suppl 8)**, S17 (2012).
23. Chen, J. *et al.* Associating microbiome composition with environmental covariates using generalized UniFrac distances. *Bioinformatics* **28**, 2106–2113 (2012).
24. Stoddard, S. F., Smith, B. J., Hein, R., Roller, B. R. K. & Schmidt, T. M. rrnDB: improved tools for interpreting rRNA gene abundance in bacteria and archaea and a new foundation for future development. *Nucleic Acids Res.* **43**, D593–D598 (2015).
25. Jian, C., Luukkonen, P., Yki-Jarvinen, H., Salonen, A. & Korpela, K. Quantitative PCR provides a simple and accessible method for quantitative microbiome profiling: *bioRxiv* (2018) doi:10.1101/478685.
26. Keystone Symposia | Scientific Conferences on Biomedical and Life Science Topics. Available at: https://www.keystonesymposia.org/18S6 (Accessed: 9 December 2019).
27. McMurdie, P. J. & Holmes, S. phyloseq: an R package for reproducible interactive analysis and graphics of microbiome census data. *PLoS One* **8**, e61217 (2013).
28. Warnes, G. R. *gplots: various R programming tools for plotting data*. (2016).
29. Brunson, J. C. *ggalluvial: Alluvial Diagrams in 'ggplot2'*. (2018).

Figure S1. Heatmap of all samples with relative abundance and estimated concentration data

A Relative abundances

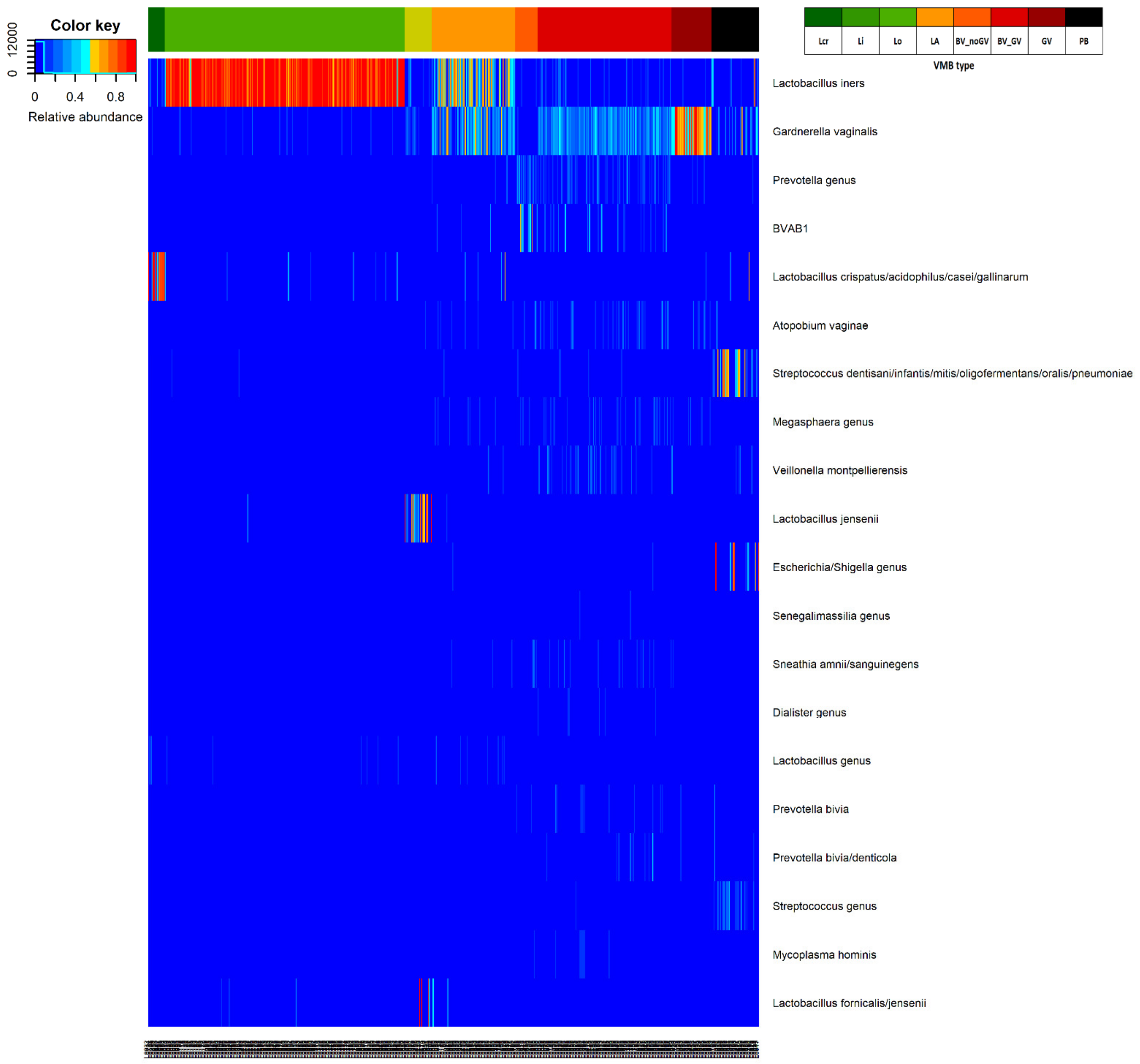

B **Estimated concentrations**

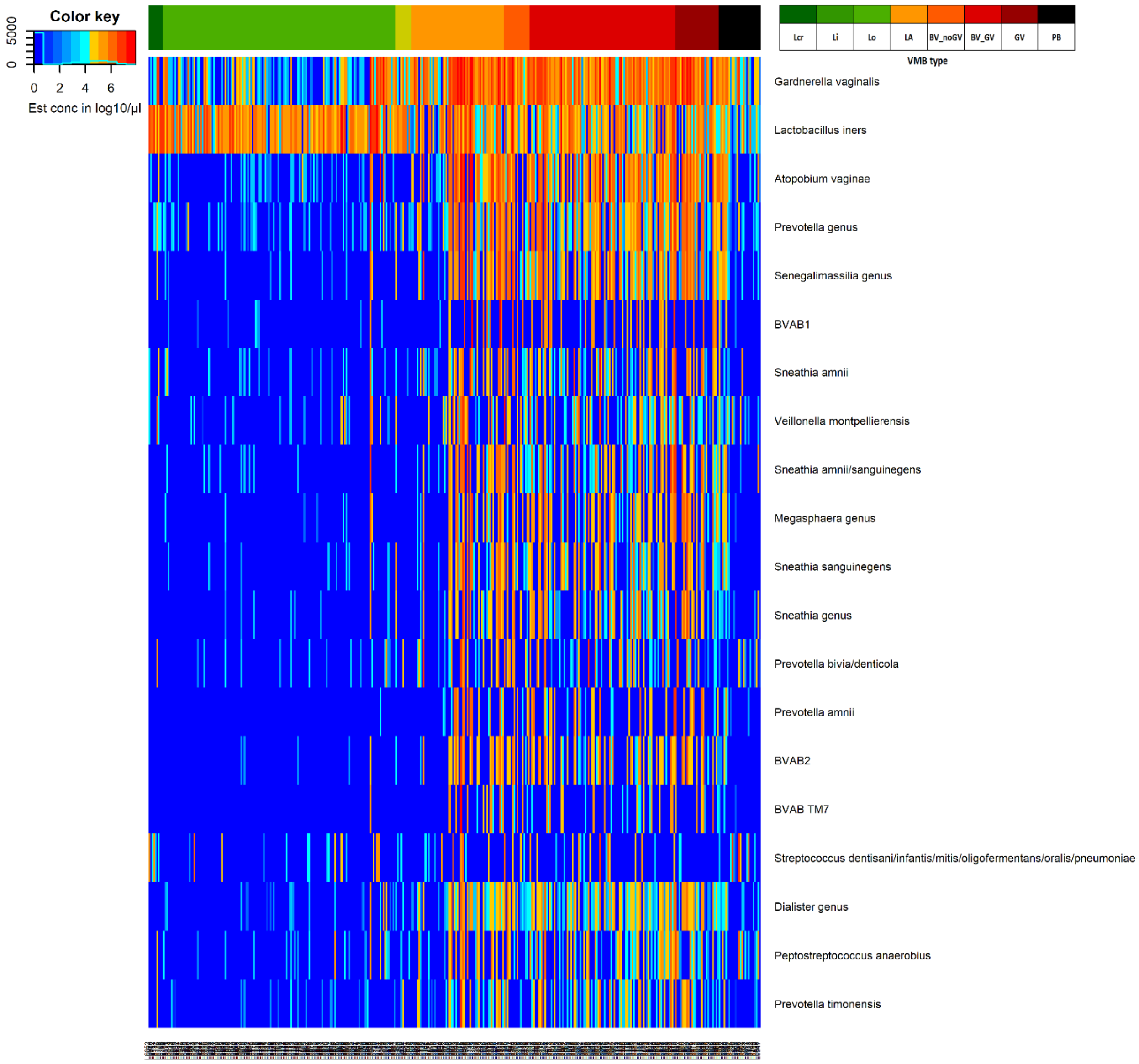

Abbreviations: BV, bacterial vaginosis; BVAB1, BV-associated bacterium type 1; BVAB2, BV-associated bacterium type 2; BVAB TM7, BV-associated bacterium (phylum TM7); BV_GV, polybacterial with ≥10% *Gardnerella vaginalis*; BV_noGV, polybacterial with <10%; est conc, estimated concentration; *G. vaginalis*; GV, *G. vaginalis*-dominated; LA, lactobacilli plus BV-anaerobes; Li, *L. iners*-dominated; Lo, other lactobacilli-dominated; PB, ≥20% pathobionts; VMB, vaginal microbiota. (**A**) Heatmap depicting all samples (n = 629) on the x-axis and the 20 amplicon sequence variants with highest mean relative abundance on the y-axis. The bar depicts VMB types. (**B**) Heatmap depicting all samples (n = 379) with valid quantitative qPCR data on the x-axis and the 20 amplicon sequence variants with highest mean 16S rRNA gene copy-normalized concentration on the y-axis. The bar depicts VMB types.

Table S1. Additional baseline characteristics

Sociodemographic characteristics at Scr/Enr	Screened (n = 175)	Enrolled (n = 68)	Controls (n = 17)	Metro (n = 17)	EF+ (n = 17)	GynLP (n = 17)	p*
Marital status, n (%)<br>- Married<br>- Never married<br>- Divorced<br>- Widowed	 8 (4.6)<br>127 (72.6)<br>34 (19.4)<br>6 (3.4)	 5 (7.4)<br>50 (73.5)<br>12 (17.6)<br>1 (1.5)	 1 (5.9)<br>16 (94.1)<br>0<br>0	 1 (5.9)<br>11 (64.7)<br>5 (29.4)<br>0	 2 (11.8)<br>10 (58.8)<br>4 (23.5)<br>1 (5.9)	 1 (5.9)<br>13 (76.5)<br>3 (17.6)<br>0	0.199
Educational level, n (%)<br>- No schooling<br>- Primary school uncompleted<br>- Primary school completed<br>- Beyond primary school	 37 (21.1)<br>72 (41.1)<br>40 (22.9)<br>26 (14.9)	 14 (20.6)<br>31 (45.6)<br>17 (25.0)<br>6 (8.8)	 5 (29.4)<br>7 (41.2)<br>4 (23.5)<br>1 (5.9)	 3 (17.6)<br>7 (41.2)<br>5 (29.4)<br>2 (11.8)	 3 (17.6)<br>13 (76.5)<br>1 (5.9)<br>0	 3 (17.7)<br>4 (23.5)<br>7 (41.2)<br>3 (17.7)	0.102
Number of sex partners last 12 months, median (IQR)	15<br>(4 – 144)	11<br>(4 – 152)	15<br>(4 – 160)	20<br>(5 – 106)	8<br>(3 – 50)	6<br>(4 – 240)	0.838
Vaginal sex frequency last 2 weeks, median (IQR)	13<br>(8 – 20)	12<br>(8 – 18)	12<br>(8 – 18)	12<br>(8 – 16)	12<br>(7 – 18)	11<br>(8 – 30)	0.975
Exchanged sex for money/ goods in past month, n (%)†	62 (93.1)	63 (92.6)	17 (100)	14 (82.4)	15 (88.2)	17 (100)	0.155
Any condom use past two weeks, n (%)<br>- Always<br>- Sometimes but not always<br>- Never	 <br>44 (25.1)<br>120 (68.6)<br>11 (6.3)	 <br>14 (20.6)<br>51 (75.0)<br>3 (4.4)	 <br>4 (23.5)<br>13 (76.5)<br>0	 <br>3 (17.6)<br>13 (76.5)<br>1 (5.9)	 <br>2 (11.8)<br>13 (76.5)<br>2 (11.8)	 <br>5 (29.4)<br>12 (70.6)<br>0	0.671
Pregnancies in lifetime, Median (IQR)†	3<br>(2 – 4)	3<br>(2 – 4)	3<br>(2 – 5)	3<br>(2 – 4)	3<br>(3 – 4)	3<br>(2 -4)	0.722
Currently breastfeeding, n (%)†	38 (22.1)	14 (21.2)	4 (23.5)	3 (18.8)	4 (23.5)	3 (18.8)	1.00
Ever washing genitalia, n (%)<br>- Yes, outside only<br>- Yes, both inside and outside‡	NA	 55 (80.9)<br>13 (19.1)	 12 (70.7)<br>5 (29.4)	 14 (82.4)<br>3 (17.6)	 15 (88.3)<br>2 (11.7)	 14 (82.3)<br>3 (17.7)	0.704
Practices to manage menstrual blood or spotting in the past 12 months, n (%)<br>- Sanitary pad<br>- Others§<br>- Nothing/no menses	NA	 <br><br>57 (83.8)<br>16 (23.5)<br>3 (4.4)	 <br><br>13 (76.5)<br>4 (23.5)<br>1 (5.9)	 <br><br>15 (88.2)<br>3 (17.6)<br>1 (5.9)	 <br><br>13 (76.5)<br>4 (23.5)<br>1 (5.9)	 <br><br>16 (94.1)<br>5 (29.4)<br>0	0.695
Frequency eating yoghurt, n (%)†,¶<br>- Never<br>- Less than once per week<br>- More than once per week	NA	 34 (50.7)<br>14 (20.9)<br>-19 (28.4)	 8 (47.1)<br>5 (29.4)<br>4 (23.5)	 7 (41.2)<br>3 (17.6)<br>7 (41.2)	 9 (52.9)<br>4 (23.5)<br>4 (23.5)	 10 (62.5)<br>2 (12.5)<br>4 (25.0)	0.792
**VMB outcomes at Enr**	**Screened (n = 175)**	**Enrolled (n = 67)**	**Controls (n = 17)**	**Metro (n = 17)**	**EF+ (n = 17)**	**GynLP (n = 16)**	**p***
RA total *Lactobacillus*, mean (95% CI)	NA	0.72<br>(0.64 – 0.80)	0.58<br>(0.38 – 0.78)	0.72<br>(0.56 – 0.88)	0.90‖<br>(0.82 – 0.98)	0.67<br>(0.51 – 0.83)	0.017
RA total BV-anaerobes, mean (95% CI)	NA	0.23<br>(0.16 – 0.30)	0.30<br>(0.12 – 0.48)	0.24<br>(0.10 – 0.39)	0.07‖<br>(-0.01 – 0.15)	0.31<br>(0.14 – 0.47)	0.015
RA total pathobionts, mean (95% CI)	NA	0.05<br>(0.02 – 0.09)	0.12<br>(-0.01 – 0.25)	0.04<br>(-0.01 – 0.09)	0.02<br>(-0.01 – 0.06)	0.02<br>(0 – 0.04)	0.303
RA total other bacteria mean (95% CI)	NA	0<br>(0 – 0)	0<br>(0 – 0.01)	0<br>(0 – 0)	0<br>(0 – 0)	0<br>(0 – 0.01)	0.034

Abbreviations: BV, bacterial vaginosis; CI, confidence interval; EF+, Ecologic Femi+; Enr, enrollment visit; GynLP, Gynophilus LP; Metro, metronidazole; RA, relative abundance. *Kruskall Wallis test, between randomization groups. †Contains between 1-4 missing values. ‡No washing or washing inside only was never reported. Twenty-seven women washed outside of menses, of whom 23 used water, three water/soap, and one 'western vaginal medicine'. §Multiple responses possible. Other practices reported were tissue, toilet paper, paper, cloth or cotton wool put inside the vagina (*n*=8) or inside the underwear (*n*=8). ¶No participants had ever used or heard of probiotics before. ‖p<0.05 by Mann Whitney U test, compared to control group.

Table S2. Antimicrobial use during the trial

Between Enr and M2	Controls<br>n women	Metronidazole<br>n women	EF+<br>n women	GynLP<br>n women	p*
Metronidazole[†]	1	2	1	3	
Tinidazole[‡]	1	0	0	0	
Both 'azoles' combined	2	2	1	3	0.688
Other antibiotic[§]	3	2	3	4	0.781
Antifungals	0	0	0	0	1.000
**Between M2 and M6**	**Controls**	**Metronidazole**	**EF+**	**GynLP**	
Metronidazole[¶]	4	1	1	2	0.439
Other antibiotic[‖]	9	6	5	6	0.634
Antifungals	0	0	0	0	1.000
**Mixed effects models using data between Enr and M2**	**Total *Lactobacillus* est conc**		**Total BV-anaerobes est conc**		
	**OR (95% CI)**	**p**	**OR (95% CI)**	**p**	
Antibiotic use for a non-study indication in the previous 14 days	0.94<br>(0.40 – 2.21)	0.879	0.68<br>(0.23 – 1.94)	0.467	

Abbreviations: BV, bacterial vaginosis; CI, confidence interval; EF+, Ecologic Femi+; Enr, enrollment visit; est conc, estimated concentration; GynLP, Gynophilus LP; M1/2, Month 1/2 visit; OR, odds ratio; TV, *Trichomonas vaginalis*. *Fisher's exact test comparing all four groups. †Prescribed for BV, TV, or amoebiasis (the latter prescribed by an external clinic). ‡Prescribed for amoebiasis/ dysentery (externally). § Includes amoxicillin prescribed for abortion prophylaxis, tonsillitis, dental caries, tooth extraction, cough, and trauma (all externally), chloramphenicol prescribed for an upper respiratory tract infection (externally), ciprofloxacin prescribed for urinary tract infection (at study clinic) and typhoid fever (externally), cloxacillin prescribed for a traumatic wound (externally), and doxycycline prescribed for chlamydia (at study clinic) and an unspecified sexually transmitted infection (externally). ¶Prescribed for BV, TV and amoebiasis (all at the study clinic). ‖Includes amoxicillin prescribed for tonsillitis and contusion (both externally), ciprofloxacin prescribed for gonorrhea or urinary tract infection (of which 8/12 at the study clinic), and doxycycline for chlamydia (all but one at the study clinic).

Figure S2. VMB comparisons between women who received metronidazole and another antibiotic at screening or metronidazole only, and between women with and without *Chlamydia trachomatis* and/or *Neisseria gonorrhoeae* at screening and enrollment

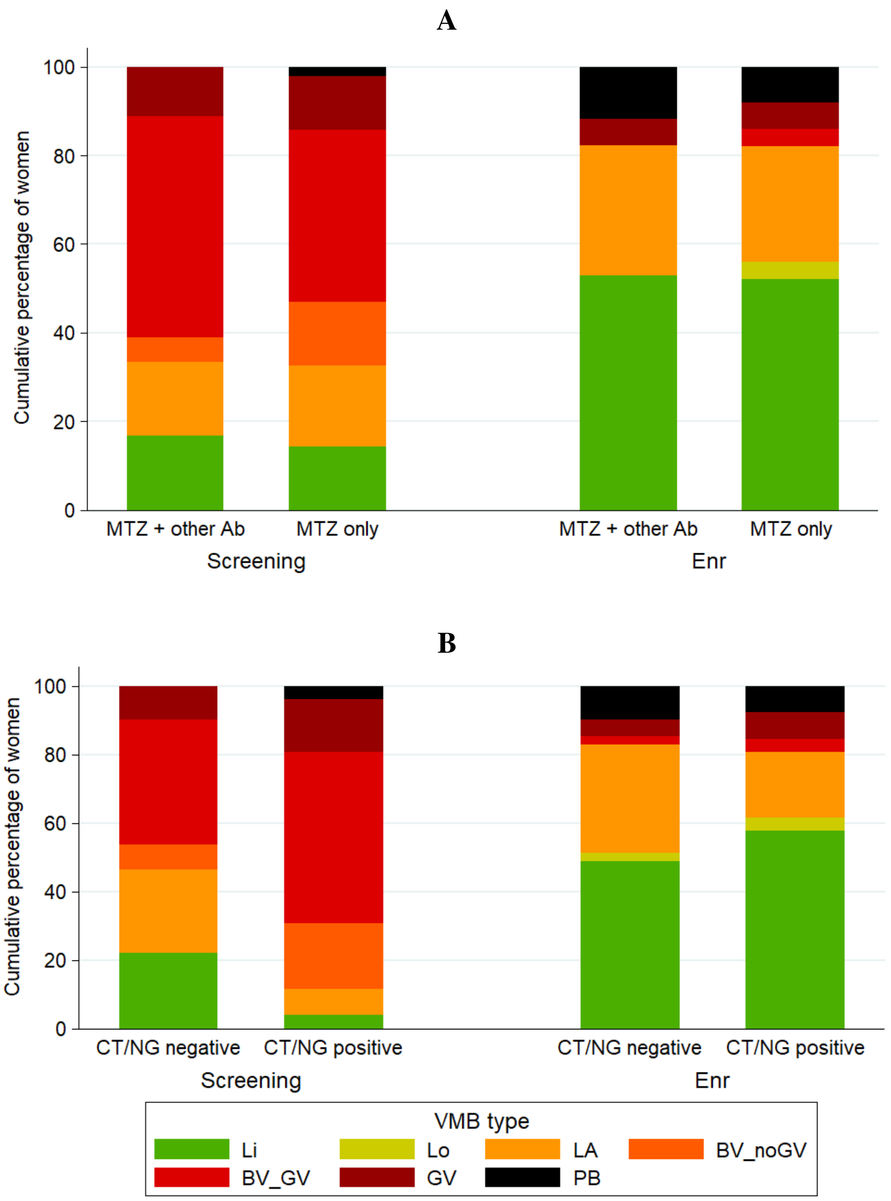

Abbreviations: Ab, antibiotics; BV, bacterial vaginosis; BV_GV, polybacterial with ≥10% *Gardnerella vaginalis*; BV_noGV, polybacterial with <10% *G. vaginalis*; CT, *Chlamydia trachomatis*; D7, Day 7 visit; Enr, enrollment visit; GV, *G. vaginalis*-dominated; MTZ, metronidazole; LA, lactobacilli plus BV-anaerobes; Li, *L. iners*-dominated; Lo, other lactobacilli-dominated; NG, *Neisseria gonorrhea*; PB, ≥20% pathobionts; VMB, vaginal microbiota. (**A**) Comparison of VMB type membership of enrolled participants who had used metronidazole with another antibiotic (n=18) or metronidazole only (n=50) between the screening and enrollment visits. (**B**) Comparison of VMB type membership of enrolled participants who were positive (n=26) or negative (n=41) for CT/NG at the screening visit, but received CT/NG treatment after the enrollment visit (if applicable). All women did use oral metronidazole for seven days between these two visits.

Table S3. Adherence with interventions during the trial

Adherence	Metronidazole (n = 17)	EF+ (n = 17)	GynLP (n = 16)
Adherence Enr–D7, median % (IQR)	100 (100 – 100)	100 (100 – 100)	100 (100 – 100)
Adherence D7–M1, median % (IQR)	100 (100 – 100)	100 (100 – 100)	100 (91.7 – 100)
Adherence M1–M2, median % (IQR)	100 (100 – 100)	100 (100 – 100)	100 (92.9 – 100)
Overall adherence Enr–M2, median % (IQR)	100 (96.3 – 100)	100 (100 – 100)	98.3 (89.3 – 100)
Overall adherence Enr–M2, n (%)			
- Perfect adherence*	12 (70.6)	10 (58.8)	8 (50.0)
- Non-perfect adherence	5 (29.4)	7 (41.2)	8 (50.0)
- Adherence ≥90%	14 (82.4)	15 (88.2)	11 (68.8)
- Adherence ≥80%	15 (88.2)	17 (100)	13 (81.3)
Number of times menses Enr–M2, n (%)†			
- Never	7 (41.2)	4 (23.5)	2 (12.5)
- Once	6 (35.3)	5 (29.4)	4 (25.0)
- Twice	4 (23.5)	8 (47.1)	10 (62.5)
- Thrice	0	0	0
Did <u>not</u> use product during menses at least once, n (%)			
- Yes	4 (23.5)	3 (17.6)	5 (31.3)
- No	6 (35.3)	10 (58.8)	9 (56.2)
- NA (never had menses)	7 (41.2)	4 (23.5)	2 (12.5)

Abbreviations: D7, day 7 visit; EF+, Ecologic Femi+; Enr, enrollment visit; GynLP, Gynophilus LP; M1/2, month 1/2 visit; NA, not applicable; IQR, inter-quartile range. *Defined as 100% of the prescribed doses used at the prescribed times after nurse review of the participant's diary card and returned used packaging and unused product. †Number of times menses in the control group: Never 2 (11.8%), once 3 (17.8%), twice 11 (64.7%), and thrice 1 (5.9%).

Table S4. Preliminary efficacy – bacterial group estimated concentrations

VMB outcome	Groups	Enr (n = 63)*	Products used			Ceased
			D7 (n = 62)*	M1 (n = 65)*	M2 (n = 63)*	M6 (n = 60)*
Estimated total 16S rRNA conc in log_{10}/µL, mean (95% CI)	Control	6.29 (5.87 – 6.72)	6.80 (6.38 – 7.23)	6.76 (6.23 – 7.30)	6.63 (6.05 – 7.21)	6.65 (6.05 – 7.25)
	Metro	6.34 (5.95 – 6.73)	6.52 (6.16 – 6.87)	6.69 (6.25 – 7.12)	6.58 (6.15 – 7.01)	6.50 (5.95 – 7.05)
	EF+	6.12 (5.86 – 6.39)	6.35 (5.93 – 6.76)	6.42 (6.03 – 6.82)	6.58 (6.26 – 6.90)	6.65 (6.28 – 7.02)
	GynLP	6.86 (6.46 – 7.26)	6.54 (6.01 – 7.06)	6.55 (5.94 – 7.16)	6.97 (6.54 – 7.40)	7.02 (6.45 – 7.60)
Estimated total bacterial conc in log_{10}/µL, mean (95% CI)	Control	5.75 (5.30 – 6.20)	6.24 (5.80 – 6.69)	6.30 (5.78 – 6.83)	6.10 (5.51 – 6.69)	6.18 (5.57 – 6.77)
	Metro	5.79 (5.39 – 6.19)	5.98 (5.63 – 6.33)	6.15 (5.70 – 6.60)	6.03 (5.59 – 6.47)	5.99 (5.34 – 6.58)
	EF+	5.54 (5.28 – 5.80)	5.77 (5.34 – 6.21)	5.86 (5.44 – 6.28)	6.05 (5.70 – 6.40)	6.18 (5.78 – 6.58)
	GynLP	6.34 (5.95 – 6.73)	6.00 (5.45 – 6.53)	6.03 (5.38 – 6.68)	6.48 (6.02 – 6.94)	6.53 (5.93 – 7.13)
Estimated total *Lactobacillus* conc in log_{10}/µL, mean (95% CI)	Control	5.22 (4.63 – 5.82)	5.15 (4.25 – 6.05)	4.81 (3.94 – 5.68)	3.86 (2.53 – 5.19)	4.86 (3.82 – 5.91)
	Metro	5.59 (5.20 – 5.97)	5.58 (5.15 – 6.01)	5.38 (4.47 – 6.29)	5.21 (4.27 – 6.14)	4.60 (3.58 – 3.62)
	EF+	5.46 (5.19 – 5.73)	5.37 (4.96 – 5.79)	5.14 (4.40 – 5.87)	5.30 (4.97 – 5.63)	5.25 (4.80 – 5.70)
	GynLP	5.97 (5.44 – 6.49)	5.55 (4.92 – 6.18)	4.93 (4.43 – 5.43)	4.68 (3.55 – 5.81)	5.05 (4.45 – 5.65)
Estimated total BV-anaerobes conc in log_{10}/µL, mean (95% CI)	Control	4.78 (4.17 – 5.39)	5.15 (4.21 – 6.09)	5.92 (5.28 – 6.55)	4.97 (3.79 – 6.15)	5.39 (5.56 – 6.22)
	Metro	4.50 (3.77 – 5.24)	4.93 (4.23 – 5.63)	4.85 (3.81 – 5.90)	4.82 (4.11 – 5.54)	5.11 (4.16 – 6.07)
	EF+	3.36 (2.43 – 4.29)	4.31 (3.51 – 5.10)	4.25 (3.26 – 5.23)	4.65 (3.47 – 5.84)	5.26 (4.18 – 6.33)
	GynLP	5.62 (4.99 – 6.25)	4.81 (3.82 – 5.79)	5.29 (4.30 – 6.28)	5.48 (4.29 – 6.67)	5.74 (4.38 – 7.09)
Estimated total pathobionts conc in log_{10}/µL, mean (95% CI)	Control	2.36 (1.28 – 3.45)	2.44 (1.08 – 3.81)	2.34 (1.13 – 3.54)	3.35 (2.21 – 4.48)	1.73 (0.69 – 2.77)
	Metro	2.09 (1.00 – 3.17)	2.62 (1.32 – 3.91)	2.37 (1.18 – 3.56)	2.87 (1.79 – 3.95)	2.20 (0.99 – 3.40)
	EF+	1.34 (0.29 – 2.39)	2.26 (1.18 – 3.35)	1.61 (0.51 – 2.72)	2.46 (1.39 – 3.54)	1.40 (0.50 – 2.30)
	GynLP	2.30 (0.98 – 3.62)	2.46 (1.30 – 3.63)	2.33 (0.99 – 3.68)	2.54 (1.24 – 3.93)	1.68 (0.25 – 3.12)
Estimated total other bacteria conc in log_{10}/µL, mean (95% CI)	Control	1.80 (0.84 – 2.76)	2.31 (1.34 – 3.29)	1.91 (0.82 – 2.99)	1.47 (0.49 – 2.46)	2.24 (1.21 – 3.26)
	Metro	1.30 (0.36 – 2.24)	1.32 (0.35 – 2.30)	1.42 (0.23 – 2.61)	1.99 (1.08 – 2.90)	2.09 (1.04 – 3.13)
	EF+	0.57 (-0.10 – 1.24)	2.73 (1.94 – 3.53)	2.62 (1.56 – 3.68)	1.48 (0.51 – 2.46)	1.66 (0.56 – 2.75)
	GynLP	2.20 (1.07 – 3.34)	2.34 (1.04 – 3.63)	2.51 (1.26 – 3.75)	2.22 (1.09 – 3.34)	2.54 (1.17 – 3.91)
Estimated total EF+ strains conc in log_{10}/µL, mean (95% CI)	Control	0.17† (-0.19 – 0.53)	0.41† (-0.19 – 1.00)	0.12† (-0.14 – 0.38)	0.16† (-0.19 – 0.51)	0.24† (-0.27 – 0.74)
	Metro	0.21† (-0.11 – 0.53)	0.39† (-0.18 – 0.97)	0.16† (-0.18 – 0.49)	0.25† (-0.28 – 0.77)	0.31† (-0.35 – 0.97)
	EF+	0.30† (-0.14 – 0.74)	1.92 (0.92 – 2.91)	1.51 (0.41 – 2.62)	0.48 (-0.22 – 1.18)	0 (0 – 0)
	GynLP	0.45† (-0.21 – 1.10)	0.79† (-0.18 – 1.76)	0.64† (-0.13 – 1.40)	0.26† (-0.30 – 0.82)	0 (0 – 0)

VMB Outcome	Groups	Enr	D7	M1	M2	M6
Estimated total GynLP strain conc in log_{10}/µL, mean (95% CI)	Control	0 (0 – 0)	0 (0 – 0)	0 (0 – 0)	0 (0 – 0)	0 (0 – 0)
	Metro	0 (0 – 0)	0 (0 – 0)	0 (0 – 0)	0 (0 – 0)	0 (0 – 0)
	EF+	0 (0 – 0)	0 (0 – 0)	0 (0 – 0)	0 (0 – 0)	0 (0 – 0)
	GynLP	0 (0 – 0)	1.05 (-0.22 – 2.31)	0.88 (-0.18 – 1.94)	0.25 (-0.28 – 0.78)	0 (0 – 0)
Difference in estimated total bacterial conc in log_{10}/µL compared to Enr visit, mean (95% CI)	Control	0	0.55 (-0.02 – 1.11)	0.52 (-0.02 – 1.06)	0.41 (-0.11 – 0.94)	0.31 (-0.35 – 0.97)
	Metro	0	0.11 (-0.12 – 0.35)	0.24 (-0.39 – 0.88)	0.21 (-0.21 – 0.62)	0.11 (-0.57 – 0.79)
	EF+	0	0.23 (-0.09 – 0.55)	0.32 (-0.08 – 0.73)	0.51 (0.06 – 0.97)	0.66 (0.26 – 1.07)
	GynLP	0	-0.27 (-0.72 – 0.18)	-0.09 (-0.65 – 0.48)	0.32 (0.01 – 0.63)	0.17 (-0.31 – 0.65)
Difference in estimated total *Lactobacillus* conc in log_{10}/ µL compared to Enr visit, mean (95% CI)	Control	0	0.07 (-0.52 – 0.66)	-0.52 (-1.21 – 0.17)	-1.03 (-2.14 – 0.09)	-0.04 (-0.72 – 0.64)
	Metro	0	-0.02 (-0.44 – 0.40)	0.13 (-0.62 – 0.88)	-0.35 (-1.46 – 0.77)	-1.03 (-2.42 – 0.34)
	EF+	0	-0.09 (-0.58 – 0.41)	-0.33 (-1.15 – 0.50)	-0.16 (-0.58 – 0.25)	-0.18 (-0.73 – 0.36)
	GynLP	0	-0.38 (-1.12 – 0.35)	-0.86 (-1.16 – -0.04)	-1.19 (-2.52 – 0.15)	-1.11 (-1.78 – -0.45)
Difference in estimated total BV-anaerobes conc in log_{10}/ µL compared to Enr visit, mean (95% CI)	Control	0	0.36 (-0.71 – 1.42)	1.10 (0.17 – 2.04)	0.18 (-0.96 – 1.32)	0.42 (-0.62 – 1.47)
	Metro	0	0 (-1.04 – 1.03)	0.03 (-1.04 – 1.11)	0.20 (-0.56 – 0.95)	0.30 (-1.09 – 1.69)
	EF+	0	0.95 (-0.04 – 1.93)	0.89 (-0.05 – 1.82)	1.30 (0.15 – 2.44)	1.98 (0.64 – 3.33)
	GynLP	0	-0.77 (-1.59 – 0.04)	-0.02 (-0.70 – 0.65)	0.03 (-1.02 – 1.08)	0.55 (-0.21 – 1.31)
Difference in estimated total pathobionts conc in log_{10}/ µL compared to Enr visit, mean (95% CI)	Control	0	0.07 (-1.87 – 2.03)	-0.16 (-1.46 – 1.15)	0.66 (-0.34 – 1.66)	-0.93 (-2.32 – 0.46)
	Metro	0	0.58 (-1.18 – 2.34)	-0.15 (-1.63 – 1.33)	0.56 (-0.90 – 2.01)	0.20 (-1.20 – 1.60)
	EF+	0	0.92 (-0.29 – 2.13)	0.27 (-0.54 – 1.80)	1.12 (-0.03 – 2.27)	-0.03 (-1.30 – 1.24)
	GynLP	0	-0.23 (-1.59 – 1.13)	0.21 (-1.38 – 1.80)	0.26 (-1.58 – 2.10)	-0.72 (-2.99 – 1.54)
Difference in estimated total other bacteria conc in log_{10}/µL compared to Enr visit, mean (95% CI)	Control	0	0.66 (-0.62 – 1.94)	0.22 (-1.09 – 1.54)	-0.48 (-1.88 – 0.92)	0.37 (-0.86 – 1.60)
	Metro	0	0.12 (-1.87 – 2.11)	-0.39 (-1.73 – 0.96)	0.49 (-0.84 – 1.81)	1.09 (-0.29 – 2.47)
	EF+	0	2.17 (1.27 – 3.06)	2.05 (1.00 – 3.11)	0.92 (-0.10 – 1.94)	1.05 (-0.22 – 2.33)
	GynLP	0	0.09 (-1.35 – 1.54)	0.45 (-0.77 – 1.67)	0.33 (-1.30 – 1.95)	0.90 (-0.39 – 2.18)

Abbreviations: BV, bacterial vaginosis; CI, confidence interval; Conc, concentration; D7, Day 7 visit; EF+, Ecologic Femi+; Enr, enrollment visit; GynLP, Gynophilus LP; M1/2/6, month 1/2/6 visit; Metro, metronidazole group; VMB, vaginal microbiota. *Total numbers are slightly lower than enrolled women (and not lost to follow-up) per time point due to invalid results. Numbers missing per group is at most two at Enr, D7, M1, or M2 visits, and four at the M6 visit. †These are naturally occurring EF+ strains with 100% identity with the EF+ probiotic strains.

Table S5. Preliminary efficacy – bacterial group relative abundances

VMB Outcome	Groups	Enr (n = 67)*	Products used: D7 (n = 64)*	Products used: M1 (n = 66)*	Products used: M2 (n = 65)*	Ceased: M6 (n = 64)*
RA total *Lactobacillus*, mean (95% CI)	Control	0.58 (0.38 – 0.78)	0.49 (0.25 – 0.73)	0.28 (0.08 – 0.48)	0.44 (0.18 – 0.70)	0.45 (0.21 – 0.68)
	Metro	0.72 (0.56 – 0.88)	0.70 (0.51 – 0.88)	0.70 (0.51 – 0.89)	0.69 (0.48 – 0.90)	0.44 (0.19 – 0.69)
	EF+	0.90 (0.82 – 0.98)	0.78 (0.60 – 0.96)	0.69 (0.47 – 0.90)	0.55 (0.32 – 0.77)	0.44 (0.22 – 0.66)
	GynLP	0.67 (0.51 – 0.83)	0.71 (0.49 – 0.93)	0.39 (0.15 – 0.63)	0.42 (0.17 – 0.67)	0.33 (0.09 – 0.57)
RA total BV-anaerobes, mean (95% CI)	Control	0.30 (0.12 – 0.48)	0.36 (0.14 – 0.57)	0.64 (0.44 – 0.84)	0.43 (0.19 – 0.67)	0.53 (0.30 – 0.76)
	Metro	0.24 (0.10 – 0.39)	0.25 (0.09 – 0.42)	0.25 (0.10 – 0.40)	0.23 (0.04 – 0.42)	0.49 (0.24 – 0.74)
	EF+	0.07 (-0.01 – 0.15)	0.19 (0.02 – 0.36)	0.26 (0.05 – 0.46)	0.38 (0.18 – 0.58)	0.56 (0.34 – 0.78)
	GynLP	0.31 (0.14 – 0.47)	0.28 (0.07 – 0.49)	0.53 (0.29 – 0.77)	0.56 (0.32 – 0.81)	0.65 (0.41 – 0.90)
RA total pathobionts, mean (95% CI)	Control	0.12 (-0.01 – 0.25)	0.15 (-0.01 – 0.31)	0.08 (0 – 0.15)	0.13 (-0.02 – 0.28)	0.02 (-0.02 – 0.06)
	Metro	0.04 (-0.01 – 0.09)	0.05 (-0.01 – 0.11)	0.05 (-0.03 – 0.12)	0.08 (-0.04 – 0.20)	0.07 (-0.04 – 0.18)
	EF+	0.02 (-0.01 – 0.06)	0.03 (0 – 0.05)	0.04 (-0.02 – 0.11)	0.07 (-0.03 – 0.16)	0 (0 – 0)
	GynLP	0.02 (0 – 0.04)	0 (0 – 0.01)	0.07 (-0.06 – 0.20)	0.01 (0 – 0.03)	0.02 (-0.02 – 0.06)
Mean RA total other bacteria, mean (95% CI)	Control	0 (0 – 0.01)	0 (0 – 0.01)	0 (0 – 0.01)	0 (0 – 0.01)	0 (0 – 0)
	Metro	0 (0 – 0)	0 (0 – 0.01)	0.01 (0 – 0.01)	0 (0 – 0.01)	0 (0 – 0.01)
	EF+	0 (0 – 0)	0 (0 – 0.01)	0.01 (0 – 0.03)	0 (0 – 0)	0 (0 – 0.01)
	GynLP	0 (0 – 0.01)	0 (0 – 0.01)	0 (0 – 0.01)	0 (0 – 0.01)	0 (0 – 0)
Difference in RA total *Lactobacillus* compared to Enr, mean (95% CI)	Control	0	-0.09 (-0.29 – 0.12)	-0.29 (-0.55 – -0.04)	-0.17 (-0.38 – 0.05)	-0.13 (-0.40 – 0.14)
	Metro	0	-0.02 (-0.24 – 0.20)	-0.01 (-0.20 – 0.19)	-0.01 (-0.21 – 0.19)	-0.26 (-0.57 – 0.05)
	EF+	0	-0.12 (-0.31 – 0.06)	-0.21 (-0.43 – 0.00)	-0.35 (-0.58 – -0.13)	-0.46 (-0.72 – -0.21)
	GynLP	0	0.02 (-0.22 – 0.25)	-0.32 (-0.60 – -0.04)	-0.27 (-0.57 – 0.02)	-0.39 (-0.68 – -0.10)
Difference in RA total BV-anaerobes compared to Enr, mean (95% CI)	Control	0	0.06 (-0.15 – 0.27)	0.34 (0.09 – 0.59)	0.17 (-0.04 – 0.38)	0.23 (-0.03 – 0.49)
	Metro	0	0.01 (-0.17 – 0.19)	0 (-0.18 – 0.19)	-0.03 (-0.24 – 0.18)	0.23 (-0.08 – 0.54)
	EF+	0	0.12 (-0.06 – 0.30)	0.18 (-0.02 – 0.39)	0.31 (0.10 – 0.52)	0.48 (0.23 – 0.73)
	GynLP	0	0 (-0.23 – 0.23)	0.26 (-0.01 – 0.52)	0.28 (-0.02 – 0.57)	0.39 (0.10 – 0.68)

VMB Outcome	Groups	Enr	D7	M1	M2	M6
Difference in RA total pathobionts compared to Enr, mean (95% CI)	Control	0	0.03 (-0.13 – 0.19)	-0.04 (-0.15 – 0.06)	0 (-0.10 – 0.09)	-0.10 (-0.19 – 0)
	Metro	0	0.01 (-0.08 – 0.09)	0 (-0.07 – 0.08)	0.04 (-0.08 – 0.15)	0.03 (-0.10 – 0.15)
	EF+	0	0 (-0.03 – 0.03)	0.02 (-0.05 – 0.09)	0.04 (-0.02 – 0.11)	-0.02 (-0.06 – 0.01)
	GynLP	0	-0.02 (-0.05 – 0.01)	0.06 (-0.08 – 0.20)	0 (-0.02 – 0.02)	0 (-0.05 – 0.05)
Difference in RA total other bacteria compared to Enr, mean (95% CI)	Control	0	0 (-0.01 – 0)	0 (-0.01 – 0.01)	0 (-0.01 – 0.01)	0 (-0.01 – 0)
	Metro	0	0 (0 – 0.01)	0 (0 – 0.01)	0 (0 – 0)	0 (0 – 0.01)
	EF+	0	0 (0 – 0.01)	0.01 (0 – 0.03)	0 (0 – 0)	0 (0 – 0.01)
	GynLP	0	0 (0 – 0.01)	0 (0 – 0.01)	0 (0 – 0)	0 (0 – 0)
RA total EF+ strains, mean (95% CI)	Control	0 (0 – 0)	0 (0 – 0)	0 (0 – 0)	0 (0 – 0)	0 (0 – 0)
	Metro	0 (0 – 0)	0 (0 – 0)	0 (0 – 0)	0 (0 – 0)	0 (0 – 0)
	EF+	0 (0 – 0)	0.02 (0 – 0.04)	0.01 (0 – 0.03)	0 (0 – 0.01)	0 (0 – 0)
	GynLP	0 (0 – 0.01)†	0.01 (0 – 0.01)†	0 (0 – 0)	0 (0 – 0)	0 (0 – 0)
RA total GynLP strains, mean (95% CI)	Control	0 (0 – 0)	0 (0 – 0)	0 (0 – 0)	0 (0 – 0)	0 (0 – 0)
	Metro	0 (0 – 0)	0 (0 – 0)	0 (0 – 0)	0 (0 – 0)	0 (0 – 0)
	EF+	0 (0 – 0)	0 (0 – 0)	0 (0 – 0)	0 (0 – 0)	0 (0 – 0)
	GynLP	0 (0 – 0)	0.02 (-0.01 – 0.06)	0 (0 – 0.01)	0 (0 – 0.01)	0 (0 – 0)

Abbreviations: BV, bacterial vaginosis; CI, confidence interval; Conc, concentration; D7, Day 7 visit; EF+, Ecologic Femi+; Enr, enrollment; GynLP, Gynophilus LP; M1/2/6, month 1/2/6 visit; Metro, oral metronidazole group; RA, relative abundance; VMB, vaginal microbiota. *Total numbers are slightly lower than enrolled women (and not lost to follow-up) per time point due to invalid results. Numbers missing per group is at most two at Enr, M1, M2, and M6 visits, and four at the D7 visit (GynLP group only, due to two missed visits). †These are naturally occurring EF+ strains with 100% identity with the EF+ probiotic strains.

Table S6. Characteristics associated with VMB composition

	Outcome variables							
	Total *Lactobacillus* est conc		Total BV-anaerobes est conc		Total patho-bionts est conc		Nugent score	
**Characteristics**	**OR (95% CI)***	**p**	**OR (95% CI)***	**p**	**OR (95% CI)***	**p**	**OR (95% CI)***	**p**
Currently uses hormonal contraception or is pregnant†	0.91 (0.60 – 1.37)	0.638	1.03 (0.61 – 1.74)	0.921	2.50 (1.36 – 4.63)	0.003	0.96 (0.40 – 2.28)	0.920
Sample taken within seven days after menses‡	0.91 (0.65 – 1.27)	0.576	1.30 (0.87 – 1.94)	0.201	0.80 (0.47 – 1.34)	0.387	2.26 (0.93 – 5.52)	0.072
Reports urogenital symptoms	0.88 (0.62 – 1.25)	0.475	3.05 (2.03 – 4.60)	<0.001	1.07 (0.63 – 1.81)	0.802	14.81 (6.64 – 33.08)	<0.001
Reports unusual vaginal discharge	0.89 (0.45 – 1.75)	0.734	4.45 (1.97 – 10.07)	<0.001	1.65 (0.59 – 4.61)	0.339	69.60 (15.14 – 320.04)	<0.001
Has taken antibiotics in the previous 14 days§	0.94 (0.40 – 2.21)	0.879	0.68 (0.23 – 1.94)	0.467	1.17 (0.87 – 11.67)	0.080	1.22 (0.10 – 14.88)	0.875
Currently breastfeeding	1.01 (0.99 – 1.03)	0.259	1.00 (0.98 – 1.02)	0.871	1.00 (0.97 – 1.03)	0.963	0.99 (0.95 – 1.04)	0.775
Uses condoms consistently	1.26 (0.93 – 1.69)	0.134	0.79 (0.55 – 1.15)	0.218	0.82 (0.52 – 1.29)	0.389	0.49 (0.24 – 0.99)	0.048
Reports five or more sex partners in the past month¶	1.24 (0.84 – 1.82)	0.276	1.00 (0.62 – 1.60)	0.986	1.94 (1.11 – 3.72)	0.019	1.01 (0.44 – 2.36)	0.973
Reports ‘below average’ sexual risk taking‖	1.14 (0.78 – 1.67)	0.506	0.98 (0.61– 1.56)	0.918	0.52 (0.29 – 0.92)	0.026	0.61 (0.24 – 1.55)	0.299
Exchanged sex for money and/or goods¶	1.42 (0.76 – 2.66)	0.271	0.63 (0.29 – 1.37)	0.243	0.77 (0.30 – 2.00)	0.598	0.53 (0.12 – 2.33)	0.401
Is 30 years or older**	1.20 (0.77 – 1.89)	0.423	0.46 (0.27 – 0.79)	0.005	0.47 (0.24 – 0.92)	0.027	0.60 (0.24 – 1.49)	0.271
Manages menses with sanitary pad (versus other methods)††	1.32 (0.75 – 2.31)	0.331	1.87 (0.61 – 5.73)	0.274	0.69 (0.16 – 1.19)	0.616	10.69 (1.13 – 101.07)	0.039
Consumes yoghurt at least once per week or more (versus never)††	0.90 (0.60 – 1.34)	0.596	1.00 (0.45 – 2.26)	0.993	0.43 (0.15 – 1.19)	0.103	3.98 (0.79 – 20.07)	0.095
Reports ever washing inside the vagina	0.92 (0.60 – 1.41)	0.692	0.74 (0.45 – 1.24)	0.253	0.99 (0.52 – 1.88)	0.964	0.47 (0.16 – 1.40)	0.174

Abbreviations: BV, bacterial vaginosis; CI, confidence interval; est conc, estimated concentration; OR, odds ratio. *Covariates were tested in mixed effects models with participant identification number as a random effect, the potential predictor as fixed effects, and the four vaginal microbiota outcomes as separate outcomes. All possible time points were included in the models. The potential predictor variables were not correlated with each other. †Women using a copper intra-uterine device were not included in this model. ‡Versus remainder of the cycle. §Only antibiotics given for reasons not associated with the primary/secondary outcomes of the study were included. ¶Or in the past two months (when asked at the M2 visit), or in the past four months (when asked at the M6 visit). ‖Composite variable of condom use consistency and number of sexual partners: women were considered low risk when they reported fewer than five sexual partners in the past month plus consistent condom use. The variable ‘exchanging sex for money and/or goods’ (next row) was not added to this composite variable because the great majority of women reported this behavior (Table 1). **Versus 29 years old or younger. ††Asked at the enrollment visit only.

Figure S3. Preliminary efficacy by bacterial group relative abundance

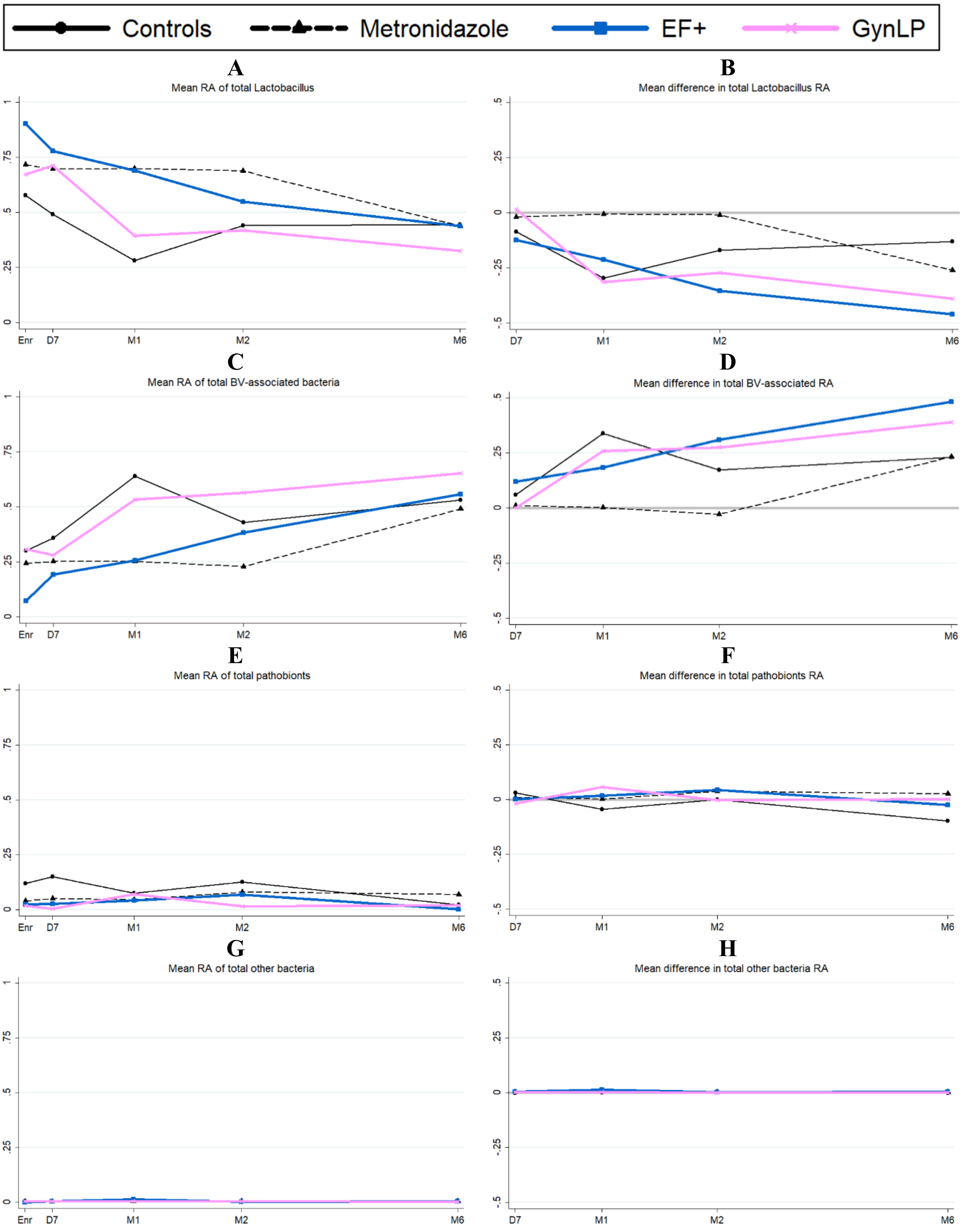

Abbreviations: BV, bacterial vaginosis; D7, Day 7 visit; EF+, Ecologic Femi+; Enr, enrollment visit; GynLP, Gynophilus LP; M1/M2/M6, month 1/2/6 visit; RA, relative abundance; Scr, screening visit.

Changes in relative abundances of bacterial groups over time per randomization group. See Table S5 for 95% confidence intervals. (**A**) Mean lactobacilli relative abundance over time. (**B**) Difference in mean lactobacilli relative abundance with enrollment, over time. (**C**) Mean BV-anaerobes relative abundance over time. (**D**) Difference in mean BV-anaerobes relative abundance with enrollment, over time. (**E**) Mean pathobionts relative abundance over time. (**F**) Difference in mean relative abundance with enrollment, over time. (**G**) Mean other bacteria relative abundance over time. (**H**) Difference in mean other bacteria relative abundance with enrollment, over time.

Figure S4. Changes in VMB type per randomization group over time

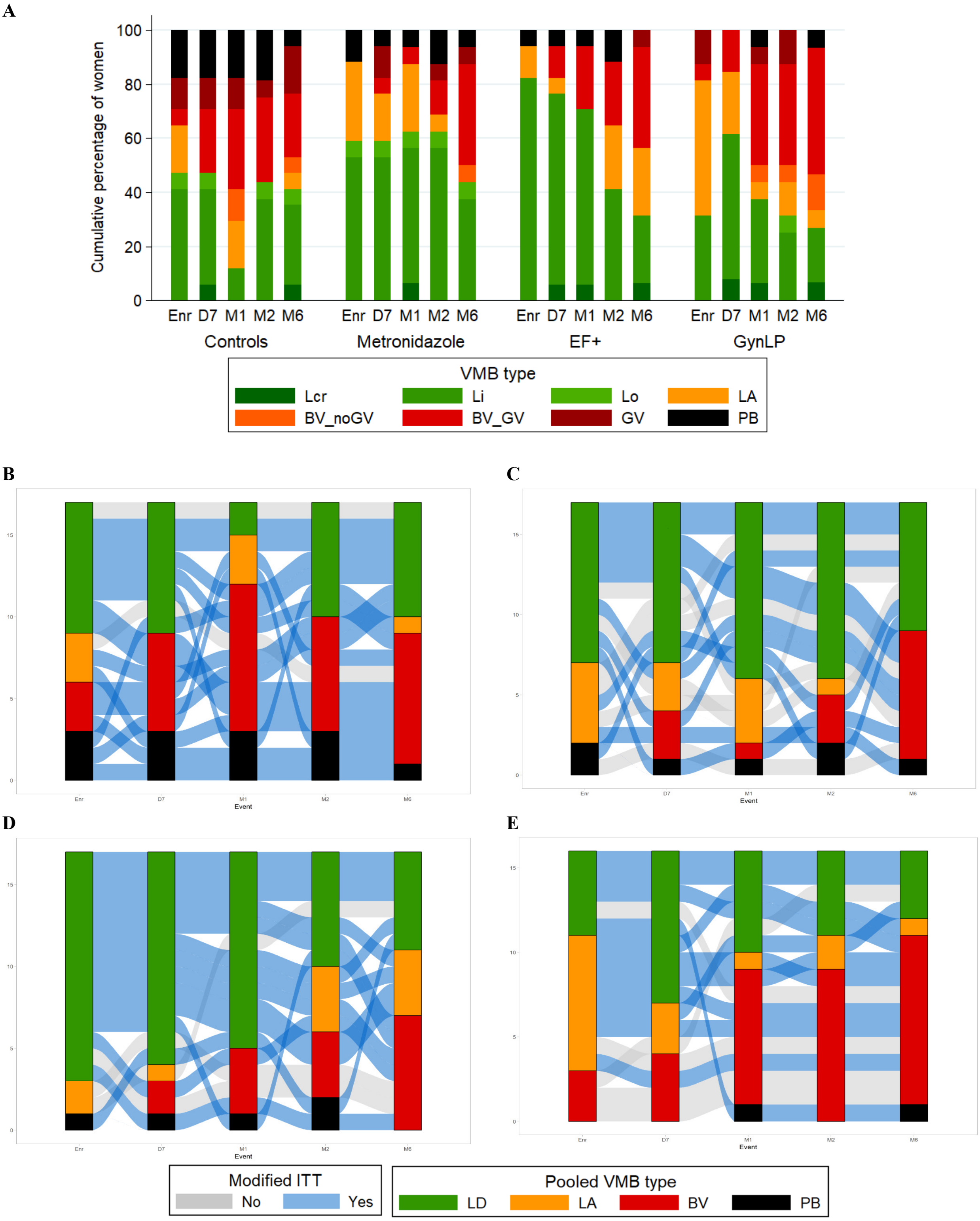

Abbreviations: BV, bacterial vaginosis-like; BV_GV, polybacterial with ≥10% *Gardnerella vaginalis*; BV_noGV, polybacterial with <10% *G. vaginalis*; D7, Day 7 visit; EF+, Ecologic Femi+; Enr, enrollment visit; GV, *G. vaginalis*-dominated; GynLP, Gynophilus LP; ITT, intent-to-treat; LA, lactobacilli plus BV-anaerobes; Lcr, *Lactobacillus crispatus*-dominated; LD, *Lactobacillus*-dominated; Li, *L. iners*-dominated; Lo, other lactobacilli-dominated; M1/M2/M6, month 1/2/6 visit; PB, ≥20% pathobionts; VMB, vaginal microbiota. (**A**) Changes in VMB type membership per randomization group over time. (**B-E**) Alluvial diagrams of changes in pooled VMB type over time per group: control group (**B**), metronidazole group (**C**), EF+ group (**D**), and GynLP group (**E**). Missing pooled VMB types at D7, M1, M2, and M6 were imputed with the types at the preceding visit, and at enrollment with the type at D7. Alluvial diagrams also show whether the participant is in the modified ITT population or not.

Table S7. Incidence of sexually transmitted and urinary tract infections

	Controls		Metronidazole		Ecologic Femi+		Gynophilus LP		Total	
	n/N*	IR (95% CI)	n/N*	IR (95% CI)	n/N*	IR (95% CI)	n/N*	IR (95% CI)	n/N*	IR (95% CI)
TV *InPouch:* Enr-M2	2/17	1.10 (0.35 – 3.41)	0/17	0	0/17	0	0/16	0	2/67	0.27 (0.09 – 0.84)
TV *InPouch*: M2-M6	1/17	0.20 (0.03 – 1.42)	2/16	0.42 (0.11 – 1.70)	0/16	0	1/15	0.23 (0.03 – 1.65)	4/64	0.21 (0.08 – 0.57)
CT: Enr-M6	1/17	0.16 (0.09 – 1.42)	2/16	0.36 (0.09 – 1.42)	2/16	0.29 (0.07 – 1.15)	1/15	0.18 (0.03 – 1.29)	6/64	0.25 (0.11 – 0.55)
NG: Enr-M6	1/17	0.14 (0.02 – 0.98)	2/16	0.28 (0.07 – 1.14)	2/16	0.29 (0.07 – 1.18)	0/15	0	5/64	0.18 (0.08 – 0.44)
UTI†: Enr-M6	2/17	0.39 (0.13 – 1.20)	2/16	0.27 (0.07 – 1.09)	1/16	0.14 (0.02 – 0.97)	2/16	0.29 (0.07 – 1.14)	7/65	0.27 (0.14 – 0.54)

Abbreviations: CI, confidence interval; CT, *Chlamydia trachomatis*; Enr, enrollment visit; IR, incidence rate; M2/6, month 2/6 visit; NG, *Neisseria gonorrhoeae*; STI, sexually transmitted infection; TV, *Trichomonas vaginalis*; UTI, urinary tract infection. Incidence rates of STIs and UTIs during study follow-up. Incidence rate ratios were not calculated due to low number of incident cases. *Number of women (n) who developed at least one incident infection during the specified time period as a proportion of the women who completed all follow-up visits in that time period (N). †One participant in the GynLP group was tested for UTI at an unscheduled visit between M2 and M6 and subsequently withdrew her informed consent.
